# Current Trends of Neutrophil Biology

**DOI:** 10.3390/ijms21239071

**Published:** 2020-11-28

**Authors:** Yoshiro Kobayashi

**Affiliations:** Toho University, Ota-ku, Tokyo 143-8540, Japan; yoshiro@biomol.sci.toho-u.ac.jp; Tel.: +81-3-3703-5248

Neutrophils are short-lived and terminally differentiated cells, and therefore, have been considered as effector cells to phagocytose pathogens and kill them or damage tissues. Neutrophils also secrete neutrophil extracellular traps (NETs) as effector cells, which has been studied extensively. Recent research, however, revealed that neutrophils are also immunomodulatory cells, that tissue neutrophils are different from peripheral blood neutrophils, and that neutrophils are capable to adapt to the surrounding environment. We, therefore, are at a stage to change our classical view on neutrophils. Special issue “Current Trends of Neutrophil Biology” (https://www.mdpi.com/journal/ijms/special_issues/Neutrophil_Biol) has been initiated to widen our knowledge on neutrophils and has gained interests from many researchers, ending up in 10 publications including five original papers and five review articles. 

Three of five papers deal with the roles of neutrophils in various disease, including gout, endotoxin-induced acute lung injury and bleomycin-induced pulmonary fibrosis. Dr. Davidsson et al. examined the role of neutrophils in gout and reported that in vivo transmigrated neutrophils are markedly primed for oxidative responses to monosodium urate (MSU) crystals and that MSU-triggered NET formation is independent of reactive oxygen species (ROS) production [1]. This adds an example showing that transmigrated neutrophils are activated as compared with resting neutrophils and that the requirement of ROS for NET formation depends on the stimuli, pointing to the multiple mechanisms for NET formation. Dr. Su et al. tested the hypothesis that neutrophil apoptosis is needed for the resolution of acute respiratory distress syndrome and restoration of tissue homeostasis [2]. They examined the suppressive effect of a mesenchymal stem cell-conditioned medium on neutrophil apoptosis in endotoxin-induced acute lung injury and its underlying mechanism. Dr. Chen, in the same group, on the other hand, examined the effect of nintedanib, a small molecule inhibitor of tyrosine kinase receptors, on bleomycin (BLM)-induced pulmonary fibrosis, a model of fibrotic pulmonary diseases [3]. Because nintedanib has both antifibrotic and anti-inflammatory activity, they separated these two activities by using a model of lung inflammation and fibrosis, which was induced by adoptive transfer of neutrophils isolated from mice injected with BLM. As a result, nintedanib reduced neutrophil infiltration into the lung, possibly by downregulating the expression of CXCR2 and very late antigen 4 and upregulating G protein-coupled receptor kinase 2 activity. 

The other two papers deal with the role of autophagy related gene 5 (*ATG5*) and the effect of oncolytic virus on neutrophils in vivo. Dr. Mroczek et al. examined the role of ATG5 in neutrophils by using genetically modified HL-60 cells, because ATG5 is known to have other functions than autophagy, such as development, cytokine secretion and apoptosis [4]. Forced overexpression of *ATG5* gene in differentiated HL-60 cells led to increases in PMA-induced ROS production and phagocytosis and a decrease in H_2_O_2_ and actinomycin d-induced apoptosis but not autophagy and NETs release. Dr. Stegelmeier et al., on the other hand, examined the effect of systemic administration of vascular stomatitis virus (VSV) on various aspects of neutrophil biology, including trafficking, maturation and antigen presentation, because oncolytic virus such as VSV is known to replicate in and destroy tumor cells while leaving normal cells unharmed and is currently being tested as a therapy for tumors [5]. As a result, VSV administration caused neutrophil migration from bone marrow to blood and then lung. They also showed that GFP-VSV administration caused major histocompatibility complex (MHC) class II expression of neutrophils positive for GFP (green fluorescent protein), suggesting possible antigen presentation by neutrophils. Although many questions remain, there may be positive or negative effects of neutrophils on oncolytic virus therapy of cancer.

Among five review articles, Drs. Baldanzi and Malerba summarized the studies on the role of diacylglycerol kinase α (DGKα) in neutrophil biology and pathology [6]. Upon neutrophil stimulation, DGKα activation is necessary for migration and a productive response. The role of DGKα in neutrophils is evidenced by its aberrant behavior in juvenile periodontitis patients, which express an inactive DGKα transcript. Dr. Galkina et al., on the other hand, reviewed two modes of exogenous killing by neutrophils, cytoneme and NETs [7]. The name cytoneme was originally offered for thin and long filopodia of embryonic cells, and cytoneme has been observed in a wide variety of organisms. The authors claimed that living neutrophils can catch microbes by a network of cytonemes, while dead neutrophils scavenge pathogens with NETs, although they appreciated that neutrophils are able to secrete NETs without cell death. Elucidation of the role of cytonemes in vivo awaits further study. Dr. Giacalone et al. reviewed neutrophil adaptation upon recruitment to the lung and proposed a future direction of therapy toward chronic inflammatory diseases in the lung, such as cystic fibrosis (CF), asthma and chronic obstructive pulmonary disease (COPD) [8]. Neutrophils can adapt to local environments upon recruitment to tissues, as exemplified by the difference between lung neutrophils and neutrophils infiltrated into skin. Neutrophils can also develop a unique inflammatory phenotype after recruitment into the CF airway lumen, where they maintain viability and exocytose their primary granules but have reduced ability to phagocytose bacteria. Dr. Fingerhut et al. summarized a large body of studies on evolution of neutrophils/heterophils, including cell number, morphology, nuclear shape, and functions [9]. Because in vivo studies are carried out using experimental animals, such as mice and rats, researchers always face the issue of how their results can be extrapolated to humans. In this regard, this review is unique in providing a comprehensive perspective over neutrophils/heterophils from various animal species. Dr. De Bondt et al. provide a comprehensive review over antigen presentation by neutrophils and the role of neutrophils in the pathogenesis of Multiple Sclerosis (MS) [10]. The latter has not been appreciated until recently. Neutrophils display a broad variety of effector functions enabling disease pathogenesis, including (1) the release of inflammatory mediators and enzymes, such as interleukin-1β, myeloperoxidase and various proteinases; (2) destruction and phagocytosis of myelin (as debris); (3) release of neutrophil extracellular traps; (4) production of reactive oxygen species; (5) breakdown of the blood–brain barrier; and (6) generation and presentation of autoantigens, although (auto)antigen presentation may not be considered as effector functions. 

As it may be noticed that no articles except for [10] deal with the effect of granular contents, I would like to introduce our study on linkage between neutrophils and estrous cycle in which opioid peptides are released to modulate the cycle [11] as such an example. Finally, I appreciate all the contributors to the special issue and hope many of you will enjoy reading these articles and develop your own ideas in the future. 
